# Factors associated with reduced risk of musculoskeletal disorders among office workers: a cross-sectional study 2017 to 2020

**DOI:** 10.1186/s12889-022-13940-0

**Published:** 2022-08-06

**Authors:** Bukhari Putsa, Wattana Jalayondeja, Keerin Mekhora, Petcharatana Bhuanantanondh, Chutima Jalayondeja

**Affiliations:** Faculty of Physical Therapy, Mahidol University, Salaya, Thailand

**Keywords:** Musculoskeletal disorders, Physical activity, Physical fitness, Sedentary behavior, Sitting, Stress, Office workers

## Abstract

**Background:**

Prolonged sitting at work should be avoided to reduce the risks of either noncommunicable diseases (NCDs) or musculoskeletal disorders (MSDs) among office workers. A short duration of breaks in sitting every hour can reduce cardiometabolic risk factors contributing to NCDs. However, the recommendation for a break from sitting at work to reduce the risks of MSDs has not been identified. Therefore, this study aimed to determine whether breaking by changing position at work, physical activity, physical fitness, stress and sleep were associated with MSDs among office workers.

**Methods:**

A cross-sectional study was conducted from 2017 to 2020. Participants aged 20–59 years and using a computer at work ≥ 4 days/week were recruited. Data were collected using an online self-reporting questionnaire for computer users and 5 domains of physical fitness tests. Odds ratio (OR) with 95% confidence interval (CI) and multivariate logistic regression were used for statistical analysis.

**Results:**

Prevalence of MSDs was 37.9% (*n* = 207/545) and the most area of complaint were the neck, shoulders and back. A nonsignificant association between physical fitness and MSDs among office workers was obtained. After adjusting for age, sex, body mass index, and comorbidity, moderate-to-vigorous intensity physical activity (MVPA) ≥ 150 min/week and sitting at work ≥ 4 h/day were MSDs risk factors (OR = 1.57, 95%CI = 1.04–2.37). Frequently changing positions from sitting to standing or walking at work every hour could reduce the risks of MSDs by more than 30%. The risks of MSDs increased among office workers who commuted by staff shuttle bus and personal car and had high to severe stress and slept < 6 h/day (1.6 to 2.4 times).

**Conclusion:**

Our findings indicated MVPA and prolonged sitting were MSD risk factors. We recommend office workers change position from sitting to standing or walking during work every hour and sleep ≥ 6 h/day to reduce risks of MSDs.

## Introduction

Musculoskeletal disorders (MSDs) are a common cause of health problems and the top 4th cause of global disability among office workers worldwide [1]. The Center for Disease Control and Prevention defined MSDs as an injury of muscles, nerves, tendons, joints and cartilage or spinal discs [2]. A high prevalence of MSDs was reported at the neck and lower back among office workers [3]. In 2018, the Division of Occupational and Environmental Diseases Thailand reported a large number of office workers presented MSDs caused by hazardous conditions in occupation and workplace environments [4]. Office workers spend many hours sitting while working with computers. Previous studies reported that office workers spent approximately 10.6 h/day sitting on workdays and non-workdays [5] and prolonged sitting at work was associated with serious health problems [6].

According to WHO guideline on physical activity (PA) and sedentary behavior (SB), all adults should to do at least 150 min/week of moderate to vigorous intensity of physical activity (MVPA) and limit the amount of time spent being sedentary by replacing with light intensity of PA [7]. All adults should undertake regular PA throughout the week for substantial health benefits.

Although many related studies have reported on the associations between PA and various health conditions, the effect of PA on MSDs among office workers remains inconclusive. A systematic review study examined 12 randomized control trials (RCTs) to determine the effects of PA intervention at work on MSDs among office workers [8]. They demonstrated robust evidence to support the effect of multidisciplinary PA intervention including nutrition and ergonomic programs on musculoskeletal pain and discomfort. However, some studies reported nonsignificant effects of PA on biopsychosocial factors inducing MSDs. Moreira et al. found a low percentage of MSDs among office workers meeting the PA recommendation by WHO. A nonsignificant association between MVPA and MSDs within the last 7 days and 12 months was illustrated [8]. Nguyen et al. found a negative correlation between standing/walking and MSDs among office workers. The results suggested that changing position from sitting to standing or walking at work may reduce the risks of MSDs among office workers. However, evidence remains lacking to identify the association between changing the positions from sitting at work and reduced risks of MSDs among office workers [9].

The WHO also strongly recommend for all adults to perform muscle-strengthening activities at moderate to vigorous intensity involving all major muscle groups on at least 2 days a week, as these provide additional health benefits [7]. To the best of our knowledge, aerobics activity including walking, running, swimming and bicycling also called endurance activity improves cardiorespiratory fitness and increased muscle strengthening activities increase muscular fitness. To promote the physical well-being of workers, the assessment of physical fitness should be performed for determining whether an individual is fit to work without risk to themselves or others [10]. However, there was no evidence to determine whether physical fitness including muscle strength, flexibility and cardiovascular fitness (CVF) are associated with MSDs occurrence among office workers.

Due to technology and digital disruption at work, large numbers of computers are used for work. All possible risk factors should be considered and controlled to promote a safe workplace. Several risk factors have been found to be associated with MSDs among office workers such as individual, work-related and psychosocial factors. Ranasinghe et al. investigated whether work-related risk factors such as workstation and job type can contribute to MSDs complaints in arms, wrists, hands, neck, shoulders and back [11]. They found that the work-related risk factor including incorrect body posture, bad work habits, daily computer usage, work overload and poor social support were significantly associated with MSDs among computer workers [11]. Several studies examined the association between psychosocial risk factors such as stress and MSDs occurrence among office workers [11–15]. Zakerian and Subramaniam found significant associations between stress at work and MS discomfort among office workers [12]. Hush et al. conducted a one-year follow-up study to determine risk factors for neck pain. They found that high psychological stress may increase the risk of neck pain [13]. The workers who experienced high-stress levels at work were prone to develop severe MSDs at the wrists, hands, shoulders and lower back [14]. Heo Y-S et al. reported that occupational exposures including physical and psychosocial risk factors were associated with sleep disturbance in both white- and blue-collar workers in Korea [15]. Although there were many related studies on the relationship between psychological stress at work and MSDs, the mechanism and threshold time remain ambiguous and inconclusive.

Based on the above evidence, identifying risk factors for MSDs among office workers remains essential to cover all possible factors in the digital era. Furthermore, the association between changing the position from sitting at work to reduce the risks of MSDs should be identified. Therefore, this study aimed to investigate whether a variety of factors including PA, SB, frequent change of position at work, physical fitness, stress level and sleep duration were associated with MSDs among Thai office workers.

## Materials and methods

### Participants

A cross-sectional study was conducted from 2017 to 2020 and 679 office workers aged between 20 to 59 years registered to participate in this study. They worked at a petroleum and telecommunication company in Thailand. The inclusion criteria included full-time employee, work experience > 1 year and using a computer/laptop > 4 days/week. Participants were excluded if they were unable to perform the MVPA and physical fitness test (PFT) caused by having severe medical conditions, (i.e., orthopedic injury, cardiovascular diseases, neurological conditions etc.), measured by the PA readiness questionnaire (PAR-Q) [16].

This study was approved by the Mahidol University Institutional Review Board (COA. No. MU-CIRB 2016/052.0004 and COA No. MU-CIRB 2018/124.1206).

Instruments.

#### The online self-reporting questionnaire on computer work-related exposure (OSCWE)

The OSCWE questionnaire was developed by Mekhora et al. [17] to identify the risk factors related to MSDs among computer users. It reported the agreement of experts and the internal consistency with the Cronbach’s alpha ranged from 0.34 to 0.93 [17]. It was available and freely accessed online via the link https://pt.mahidol.ac.th/project/ergo/question_en_full.php. It consisted of 30 items in five domains including personal, work-related, work environment, physical health and psychological domains. This study selected 16 items to answer our research questions as listed below.

1. Demographics included eight items in personal, work-related and physical health domains: age, sex, weight change over the past two years, working experience in the current workplace (years), monthly income, comorbidity and current smoking and alcohol consumption. For comorbidity, participants were asked, *“Do you have any other health problems apart from MSDs, e.g., hypertension, hyperlipidemia, diabetes, respiratory problems or cancer?*
2. PA and SB included five items in personal and work-related domains. PA comprised the amount of MVPA (minutes/week) and commuting modes. Regarding MVPA, the type, duration and frequency of PA during the last seven days were collected. The questions consisted of, *“Did you perform moderate to vigorous intensity PA during the last seven days? Please specify type, duration per session and frequency per week?”* For commute modes from home to work, participants were asked to choose a usual mode of commuting such as public transportation, employee shuttle bus or personal vehicle. For SB, the questions included, *“How many hours/day do you spend sitting at work, i.e., use computer, meeting *etc*.” “How many hours/day do you use a computer or a mobile device during leisure time?”* and “*Do you change your posture at least once an hour while working with a computer?”* These items represented the amount of time in sitting at work (hours/day), screen time use of computer for recreation at home (hours/day) and frequency of changing position every hour at work (yes/no).
3. Stress level and sleep duration were in psychological and physical health domains. Stress was assessed using the Suanprung Stress Test 20 (SPST-20) which asked participants to rate their stress level using a 5-point Likert scale for 20-items. The scale ranged from 1 (no stress) to 5 (severe stress) and total score was 100. The SPST-20 had an acceptable reliability from the Cronbach’s alpha of 0.7 [18]. In this study stress was categorized in normal (≤ 24 scores), moderate (25–42 scores) and high to severe stress (≥ 43 scores). Sleep duration asked, *“Do you sleep less than six hours/day?* The answer of yes/no was categorized in sleep ≥ 6 h/day and < 6 h/day.
4. MSDs were in the physical health domain and the question was, *“In the past seven days, did you have any pain or injuries of the bones, joints, ligaments or muscles? If yes, please indicate the area that bothered you the most with pain level and symptoms”*. This criterion was used to classify participants with having MSDs based on the self-report of the most pain in the body area within the past 7 days [3, 11, 19]. Participants who answered “yes” were classified as having MSDs and those who answered “no” were classified as not having MSDs.

#### Physical Fitness Test (PFT)

Before the PFT, all participants were screened by blood pressure (BP). Those who had BP > 120/80 mmHg were not allowed to perform the YMCA three-minute step test and trunk endurance tests. PFT was assessed by well-trained physical therapists and the tests are listed below.

1. Body compositions comprised body mass index (BMI), waist circumference (WC), and body fat. BMI was calculated by body weight and height (kg/m^2^). According to the WHO guidelines of cardiometabolic risk factors for Asian populations [20], BMI was divided in three levels: BMI < 23.0 kg/m^2^, BMI = 23.0–27.5 kg/m^2^ and BMI > 27.5 kg/m^2^. WC was measured in the horizontal plane at the narrowest area of the midway between the lowest ribs and the iliac crest using a tape measure [21]. The percent of body fat was measured by bioelectrical impedance analysis (BIA) (Omron® HBF-500 BIA scale).
2. CVF was measured using the YMCA three-minute step test. Participants were asked to step up and down a box (30 cm in height) for three minutes following the beat by a metronome (96 beats per minute or stepping rate of 24 steps per minute). Heart rate (HR) at one minute after completing the test was recorded [22].
3. For muscular strength, deep neck muscle strength was assessed using the craniocervical flexion test (CCFT) [23]. Participants lay down on a bed and were asked to perform “chin in” for ten seconds and repeated ten times in five different levels. Each level of pressure was set by a pressure biofeedback unit (PBU). A 30-s rest was provided between each level. A performance index was calculated, and the highest index score was 100 [23]. Moreover, grip strength was assessed by hand-held dynamometer. Participants were asked to bend their elbows at 90 degrees and squeeze a hand-held dynamometer with maximum effort for three to five seconds, three trials and one-minute rest were provided between each trial. The highest score was recorded for data analysis [24].
4. For muscular endurance, deep cervical flexor muscle endurance was assessed using the neck endurance test [25]. Participants were asked to performed “chin-in” and lift their head up. Time was recorded until they could not hold this position, or their head dropped from the chin in or their head rested on the assessor’s hand. The participants could stop the test anytime if they felt pain or discomfort. Back extensor muscle endurance was assessed using the Ito’s test [26]. Participants were timed after they lifted their upper trunk off the floor from a prone lying position. The maximum time was 300 s and they could stop the test anytime if they felt pain or discomfort.
5. The flexibility test of the back and legs was measured using the sit and reach test and the modified Schober’s test. For the sit and reach test, participants were asked to sit with legs extended and feet against the base of the sit-and-reach box, place one hand on top of the other, then slowly reach forward as far as they could, holding this position for two seconds. The assessor recorded the length in cm [27]. The modified Schober’s test was established to measure lumbosacral spine mobility. Participants were asked to stand, and assessors drew the first line at the lumbosacral junction location between the posterior superior iliac spine (PSIS) and the second line was marked at 10 cm above the first line and the third line was marked at 5 cm below the first line. Participants were asked to bend forward as far as they could in the direction to touch their toes. The new distance between the first line and the second line was measured. Lumbosacral mobility was reported as the difference between this measure and the initial distance of 15 cm [28].

### Statistical analysis

Statistical data analysis was performed using the software, Statistical Package for the Social Sciences (SPSS®) (Version 23.0; IBM, Armonk, NY, USA). The categorical data were reported in number and percentage (%) of the total population. The continuous data were reported in mean and standard deviation (SD) of the PFT score. To be clearly comparable with related studies and public health implementation, our study categorized four continuous variables for data analysis: age groups (20 to 29 years, 30 to 39 years, 40 to 49 years and 50 to 59 years), BMI (BMI < 23.0 kg/m^2^, 23.0 ≤ BMI ≤ 27.5 kg/m^2^ and BMI > 27.5 kg/m^2^), MVPA (≥ 150 min/week and < 150 min/week) and sitting at work (≥ 4 h/day and < 4 h/day). The prevalence of MSDs within seven days was calculated by dividing the number of persons with MSDs within 7 days by the total number of office workers who participated and it was presented as a percentage (%). Binary logistic regression was used to analyze the association between risk factors and presence of MSDs (yes/no). The Odds Ratio (OR) and 95% confidence interval (CI) were calculated to represent the strength of association between each risk factor and MSDs. An OR greater than 1.0 represents a risk factor of MSDs. Each risk factor was entered for the analysis including MVPA, sitting time at work, frequency of changing position at work, commute from home to work, stress, and sleep time each day. To minimize the effect of confounding factors, age, sex, BMI, and comorbidity were included in adjusted analyses. The adjusted OR with 95% CI was identified using multivariate logistic regression. A level of significance was set at *p*-value < 0.05.

## Results

**Fig. 1:**
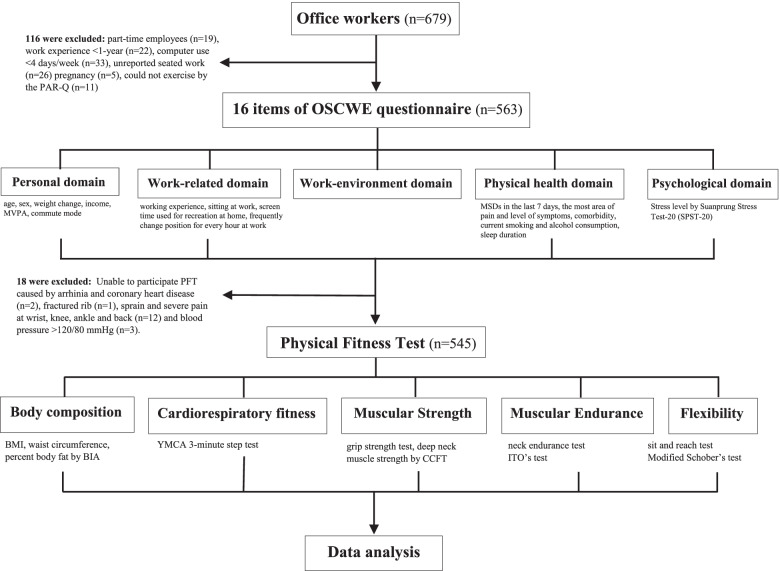
Flowchart for data collecting process

Of 679 who registered in this study, 116 office workers were excluded because they did not meet the inclusion criteria. Of 563, 18 participants were unable to perform PFT due to having arrhythmia and coronary heart diseases (*n* = 2), fractured rib (*n* = 1), sprain and severe pain at the wrist, knee, ankle and back (*n* = 12) and blood pressure > 120/80 mmHg (*n* = 3). Therefore, 545 participants completed the OSCWE questionnaires and PFT and their data were used for analysis. A flowchart of data collection is shown in Fig. 1.

The prevalence rate of MSDs within 7 days was 38% (n = 207/545) in Thai office workers. Office workers presenting MSDs reported the most areas of pain at the neck, shoulders and lower back. The average visual analog scale was greater than 5 but did not disturb or cause absence from work. Prevalence of MSDs were 27.5% at the neck (*n* = 150/545), 22.7% at the shoulders (*n* = 124/545) and 17.6% at the lower back (*n* = 96/545).

Table 1 presents the demographics and MSDs in among 545 office workers.

**Table 1:** Demographics and musculoskeletal disorders (MSDs) among office workers (*n* = 545)

Demographics	Total (*n* = 545)	MSDs (*n* = 207)	Prevalence rate (%)	OR	95% CI	*p*-value
**Age (year)**						
20–29 years	80	27	33.7	1.00	-	-
30–39 years	242	86	35.5	1.09	0.64, 1.86	0.736
40–49 years	163	67	41.1	1.37	0.78, 2.39	0.269
50–59 years	60	27	45.0	1.65	0.83, 3.30	0.152
**Sex**						
Male	212	77	36.3	1.00		
Female	333	130	39.0	1.11	0.77,1.59	0.561
**BMI (kg/m**^**2**^**)**						
BMI < 23.0	294	98	33.3	1.00	-	-
23.0 ≤ BMI ≤ 27.5	178	68	38.2	1.22	0.82, 1.79	0.320
BMI > 27.5	73	41	56.1	2.53	1.49, 4.25	< 0.001^**^
**Weight change in 2 years**						
Weight stable	166	50	30.1	1.00	-	-
Weight loss	87	32	36.7	1.33	0.76, 2.29	0.312
Weight gain	292	125	42.8	1.72	1.14, 2.57	0.009^*^
**Working Experience (year)**						
1–3 years	31	14	45.1	1.00	-	-
4–6 years	62	18	29.0	0.43	0.17, 1.09	0.076
7–10 years	211	79	37.4	0.64	0.29, 1.41	0.272
> 10 years	241	96	39.8	0.70	0.32, 1.53	0.384
**Income (THB/month)**						
up to 30,000	68	28	41.1	1.00	-	-
30,001 – 50,000	86	29	33.7	0.72	0.37, 1.40	0.342
50,001 – 100,000	198	78	39.3	0.93	0.53, 1.64	0.818
more than 100,000	193	72	37.3	0.86	0.49, 1.52	0.613
**Comorbidity**						
No	483	172	35.6	1.00	-	-
Yes	62	35	56.4	2.32	1.39, 3.96	0.002^*^
**Current smoking**						
No	514	199	38.7	1.00	-	-
Yes	31	8	25.8	0.54	0.23, 1.24	0.149
**Alcohol consumption**						
< 3 times/week	524	200	38.1	1.00	-	-
≥ 3 times/week	21	7	33.3	0.80	0.31, 2.02	0.641

^*^p-value < 0.05, ^**^p-value < 0.001

*Abbreviations*: *BMI* Body mass Index, *THB* Thai Baht

The prevalence rate of MSDs was higher among older age than younger age subjects (49.0% for 50 to 59 years, 41.1% for 40 to 49 years, 35.5% for 30 to 39 years and 33.7% for 20 to 29 years). Office workers presenting BMI > 27.5 kg/m^2^ were twice as likely to have MSDs as those presenting BMI < 23.0 kg/m^2^ (OR = 2.53, 95%CI 1.49–4.25, *p* < 0.001). The probability of MSDs occurrence increased among office workers with weight gain in two years (OR = 1.72, 95%CI 1.14–2.57, *p* = 0.009) and comorbidity (OR = 2.32, 95%CI 1.39–3.96, *p* = 0.002).

Table 2 presents the association of PA, SB and MSDs among office workers. After adjusting for age, sex, BMI, and comorbidity, the results demonstrated that office workers having MVPA ≥ 150 min/week were more likely to have MSDs compared to those having MVPA < 150 min/week (adjusted OR = 1.64, 95%CI 1.09–2.45, *p* = 0.015). The office workers having sitting at work ≥ 4 h/day were 2.51 times more likely to have MSDs when compared to those who sit at work < 4 h/day (adjusted OR = 2.51, 95%CI 1.08–5.82, *p* = 0.032). When combine both PA and SB, the office workers having MVPA ≥ 150 min/week and sitting at work ≥ 4 h/day were at high risk of MSDs when compared with those having less MVPA and sitting time (adjusted OR = 1.57, 95%CI 1.04–2.37, *p* = 0.030). The office workers not frequently changing position from sitting to standing or walking were more likely to experience risk of MSDs than those who did (adjusted OR = 1.47, 95%CI 1.03–2.10, *p* = 0.034).. For commuting from home to work, office workers who commuted by shuttle bus or personal car/motorcycle were more likely to have MSDs than those who commuted using public transportation (adjusted OR = 1.74, 95%CI 1.08–2.80, *p* = 0.022).

**Table 2:** Physical activity (PA) and sedentary behavior (SB) associated with musculoskeletal disorders (MSDs) among office workers

PA and SB	Have MSDs (*n* = 207)		Not have MSDs (*n* = 338)		Unadjusted			Adjusted by age, sex, BMI, and comorbidity		
	**n**	**%**	**n**	**%**	**OR**	**95% CI**	***p*-value**	**OR**	**95% CI**	***p*-value**
**MVPA (*n* = 457)**										
< 150 min/week	101	56.1	188	67.9	1.00	-	-	1.00	-	-
≥ 150 min/week	79	43.9	89	32.1	1.65	1.12, 2.44	0.011*	1.64	1.09,2.45	0.015^*^
**Sitting at work (*n* = 543)**										
< 4 h/day	8	3.9	27	8.0	1.00	-	-	1.00	-	-
≥ 4 h/day	199	96.1	309	92.0	2.18	0.97, 4.89	0.059	2.51	1.08,5.82	0.032^*^
**MVPA & Sitting at work (*n* = 457)**										
MVPA < 150 min/wk & Sit ≥ 4 h/d	97	46.8	172	50.8	1.00	-	-	1.00	-	-
MVPA < 150 min/wk & Sit < 4 h/d	4	1.9	16	4.7	0.44	0.14,1.36	0.156	0.38	0.12,1.21	0.103
MVPA ≥ 150 min/wk & Sit < 4 h/d	3	1.4	5	1.5	1.06	0.25,4.55	0.933	0.99	0.21,4.66	0.994
MVPA ≥ 150 min/wk & Sit ≥ 4 h/d	76	36.7	84	24.8	1.60	1.07,2.38	0.020^*^	1.57	1.04,2.37	0.030^*^
**Screen use for recreational at home (*n* = 545)**										
< 4 h/day	94	45.4	172	50.9	1.00	-	-	1.00	-	-
≥ 4 h/day	113	54.6	166	49.1	1.25	0.88,1.77	0.203	1.31	0.91, 1.88	0.134
**Frequently change position at work (*n* = 543)**										
Yes, every hour	105	50.7	201	59.4	1.00	-	-	1.00	-	-
Not frequent	102	49.3	134	39.4	1.45	1.02, 2.06	0.035^*^	1.47	1.03, 2.10	0.034*
**Commute from home to work (*n* = 480)**										
Public transportation	31	15.0	80	23.6	1.00	-	-	1.00	-	-
Staff shuttle bus and personal car	152	73.4	217	64.2	1.81	1.13,2.87	0.012^*^	1.74	1.08, 2.80	0.022*

^*^p-value < 0.05

*Abbreviation*: *MVPA* Moderate to vigorous intensity of physical activity

Table 3 presents the association between physical fitness and MSDs among office workers (*n* = 545). The results showed that BMI (adjusted OR = 1.07, 95% CI = 1.02–1.11) was significantly associated with MSDs among office workers. For the other tests of PFT, nonsignificant associations were observed between the test and MSDs.

**Table 3:** Physical fitness associated with musculoskeletal disorders (MSDs) among office workers (*n* = 545)

Physical Fitness	Have MSDs (*n* = 207)		Not have MSDs (*n* = 338)		Unadjusted			Adjusted by age, sex, BMI and comorbidity		
	**n**	**mean ± SD**	**n**	**mean ± SD**	**OR**	**95% CI**	***p*-value**	**OR**	**95% CI**	***p*-value**
**Body Mass Index (kg/m**^**2**^**)**^a^	207	24.07 ± 4.46	338	22.75 ± 4.21	1.07	1.02, 1.12	< 0.001^**^	1.07	1.02, 1.11	0.002^*^
**Waist circumference (cm)**	202	80.73 ± 12.32	330	78.83 ± 11.93	1.01	0.99, 1.02	0.080	1.00	0.98, 1.02	0.498
**Body fat (%)**	201	28.20 ± 6.63	331	26.71 ± 6.68	1.03	1.00, 1.06	0.014^*^	1.02	0.98, 1.05	0.238
**Heart Rate at 1 min (bpm)**^b^	183	105.03 ± 17.95	311	103.32 ± 18.30	1.00	0.99, 1.01	0.314	1.00	0.99, 1.01	0.698
**Grip strength (kg.)**										
Right hand	191	26.36 ± 8.47	321	26.39 ± 8.30	1.00	0.97, 1.02	0.976	1.00	0.97, 1.02	0.945
Left hand	191	24.84 ± 7.72	321	25.15 ± 8.07	0.99	0.97, 1.02	0.667	0.99	0.96, 1.02	0.784
**Neck strength by the CCFT**	186	65.00 ± 44.89	314	72.12 ± 62.85	0.98	0.99, 1.00	0.182	0.99	0.99, 1.00	0.203
**Neck endurance (sec.)**	185	37.84 ± 21.74	320	38.84 ± 23.50	0.99	0.99, 1.00	0.634	0.99	0.98, 1.00	0.610
**Back endurance by ITO’s test (sec.)**	145	138.91 ± 73.37	258	139.02 ± 70.42	1.00	0.99, 1.00	0.988	1.00	0.99, 1.00	0.912
**Flexibility of Back (cm.)**										
by sit and reach test	152	0.23 ± 10.56	270	-0.03 ± 10.89	1.00	0.98, 1.02	0.809	1.00	0.98, 1.02	0.895
by modified Schober’s test	152	5.10 ± 1.20	269	5.19 ± 1.43	0.95	0.81, 1.10	0.489	094	0.80, 1.09	0.442

^*^p-value < 0.05, ^**^p-value < 0.001

*Abbreviations*: *CCFT* The cranio-cervical flexion test

^a^The OR, 95%CI and statistics were adjusted by age, sex and comorbidity

^b^measured by the 3-min step test

Table 4 presents the association between stress level, sleep hours/day and MSD occurrence. The risk of having MSDs increased among office workers reporting high to severe stress levels. The odds of having MSDs among workers who had high to severe stress (SPST-20 > 43 scores), were 2.63 times (95% CI 1.52–4.55, *p* < 0.001) higher than those reporting normal stress levels. A high risk of MSDs was also found among those who had a moderate stress level (OR = 1.45, 95% CI 0.88–2.39) compared with normal stress level. For sleep duration, a high risk of MSDs was also found to be significantly associated with sleep time. Office workers reporting a duration of sleep < 6-h daily were two times more likely to have MSDs than those who slept ≥ 6-h daily (adjusted OR = 1.60, 95%CI 1.11–2.32, *p* = 0.012) after adjusting for age, sex, BMI and comorbidity.

**Table 4:** Stress and Sleep time associated with musculoskeletal disorders (MSDs) among office workers

Stress and sleep	Have MSDs (*n* = 207)		Not have MSDs (*n* = 338)		Unadjusted			Adjusted by age, sex, BMI, and comorbidity		
	**n**	**%**	**n**	**%**	**OR**	**95% CI**	***p*-value**	**OR**	**95% CI**	***p*-value**
**Stress level (*n* = 540)**										
Normal stress (< 24)	29	14.0	75	22.5	1.00	-	-	1.00	-	-
Moderate stress (25–42)	112	54.1	193	58.0	1.50	0.92, 2.44	0.103	1.44	0.88, 2.37	0.152
High to severe stress (≥ 43)	66	32.0	65	19.5	2.63	1.52, 4.55	< 0.001^**^	2.41	1.36, 4.27	0.002*
**Sleep time a day (*n* = 545)**										
≥ 6 h/day	119	57.5	235	69.3	1.00	-	-	1.00	-	-
< 6 h/day	88	42.5	103	30.7	1.67	1.16, 2.38	0.006*	1.60	1.11, 2.32	0.012*

^*^p-value < 0.05, ^**^p-value < 0.001

## Discussion

Our results demonstrated that the prevalence of MSDs within seven days was 37.9% among Thai office workers. The neck, shoulders and back were identified as the most common areas of complaint during work. The prevalence of MSDs in this study were in the range reported in previous studies varying from 33 to 65% [3, 29–33]. However, the prevalence of MSDs categorized by body area were 27.5%, 22.7%, and 17.6% at the neck, shoulders, and lower back respectively which a lower prevalence of these areas than presented in the previous studies. This was due to differences in methods used to observe including time to recall MSDs, time exposed to computer work and type of occupations.

Our findings indicated that PA and SB were associated with MSD occurrence among office workers and are similar to previous studies. Prolonged sitting has been associated with many of health and chronic disease risks [34]. A recent systematic review with meta-analysis reported that occupational SB was associated with MS pain, discomfort and disability. Dzakpasu et al. demonstrated evidence to support significant associations between workplace sitting time and MSD in the neck, shoulders and lower back among office workers. This could be explained by static sitting posture for long periods of time that may produce tension, strain and fatigue in the muscles inducing MS pain and discomfort, and other chronic conditions [35]. Jun et al. reviewed many prospective studies and found strong evidence demonstrated sitting for computer work ≥ 4 h/day was a risk factor for neck and shoulder pain among office workers (relative risk = 1.36, 95% CI 1.10–1.88) [36]. Therefore, continuous sitting for work without a break posed a risk factor of MSDs among office workers.

Our findings revealed that office workers frequently changing position at work were less likely to have MSDs than those who did not. The office workers who reported changing posture from sitting to standing or walking every hour demonstrated low risk of MSDs occurrence. The results were similar to the systematic reviews conducted by Waongenngarm et al. [37]. They presented strong evidence to support breaks from sitting by changing posture to minimize the cause of musculoskeletal pain and discomforts by prolonged sitting. However, duration of breaks varied from 5 min to 2 h. Balci and Aghazadeh demonstrated that frequent short duration breaks every hour significantly decreased MS discomfort among office workers [38]. Jalayondeja et al. also suggested office workers take breaks from sitting during work to reduce the risk of NCDs and cardiometabolic risk factors (CMRFs) [39]. Office workers should perform short duration from two to five minutes with active break more than twice daily to promote health benefits and prevent all-cause mortality. Based on the above evidence, our study recommended office workers should avoid prolonged sitting for work and perform frequent short break by changing their posture from sitting to standing or walking every hour to reduce risks of MSDs, NCDs and CMRFs.

Those who commute from home to work by public transportation exhibited lower risks of MSDs than those commuting by other forms of transportation to work such as the shuttle bus of the company, personal car or personal motorcycle. One explanation for this is increased PA associated with using public transportation. For example, Rissel et al. reviewed 27 studies and suggested that physical activity was part of public transportation use the same as walking or bicycling. People who commuted by public transportation had to walk greater than 30 min to a public transit stop compared with 8 min for walking to private transport [40].

For stress and sleep duration, we found significant associations between high to severe stress and MSDs. Sleep duration < 6 h/day was associated with the occurrence of MSDs and corresponded to Chun et al. who reported significant decreases of MSDs among Korean people who slept approximately 5 to 7 h/day [41]. Moreover, Strine and Hootman reported that sleep problems or insomnia or trouble falling asleep was associated with low back and neck pain among Americans [42]. The mechanism of association between sleep duration and MSDs was explained by Kundermann et al. and Edwards et al. [43, 44]. They concluded that sleep deprivation or insufficient sleep time could increase the sensitivity of noxious stimuli and decreased endogenous pain inhibitory processes. Sleeping less than six hours/day related to high levels of pain threshold among people with musculoskeletal pain [44]. Many previous studies [45–47] found that work exposure to high physical and psychosocial demands combined with long periods of computer work without insufficient breaks or recovery time could induce increases in muscle tension and fatigue that can contribute to the development of MSDs. High job demand combined with low job control were considered an important factor associated with MSDs among office workers [48]. High job demand, low skill discretion, low decision and low social support combined with long duration of computer use were significantly associated with neck pain among workers [49].

However, unexpected findings were demonstrated in our study. First, higher odds of having MSDs was found among office workers performing MVPA ≥ 150 min/week (adjusted OR = 1.64, 95%CI = 1.08–2.45) when compared with MVPA < 150 min/week. Our finding did not contrast with the previous studies and WHO for PA promotion to improve health benefits. However, we believe that the effect of interplaying between PA and SB should be pooled for health risk identification rather than regarding each effect. With this approach, our results demonstrated the combined effect of MVPA and sitting time on MSDs among office workers. The risk for MSDs was reduced among those who engaged in MVPA > 150 min/week of MVPA and < 4 h/day for sitting at work. In a previous study, four mutually exclusive categories of PA and SB demonstrated different biomarkers concerning health [50]. Bakrania et al. defined individual behavior by MVPA 150 min/week and sedentary time. Three types behaviors: Busy Bee, Sedentary Exerciser and Light Movers were more likely to reduce cardiometabolic risk factors when compared with Couch Potato [50]. Sedentary exercisers, or those who are physically active (MVPA ≥ 150 min/week) but sat at work for long periods daily (> 4 h/day), might experience risk of either NCDs and MSDs. As a consequence, daily balanced behavior between PA and SB should be considered to prevent NCDs and MSDs.

Secondly, nonsignificant associations were observed between PFT and MSDs in this study. In contrast with our hypothesis, muscle strength, endurance and flexibility and cardiovascular endurance were not associated with MSD occurrence among office workers. Muscular strength and endurance are well known and good predictors for health, mobility and functional demands in daily living tasks [51]. Although these tests can be used as an indicator of functional and physical capacities, the PFT might not be appropriate for low physical demanding work such as that of office workers. Similarly, Multanen et al. [52] also reported no associations between neck muscle strength or neck range of motion and neck pain and disability. They suggested that screening for neck muscle weakness or flexibility in healthy individuals was not recommended to prevent MSDs. Hamberg-van Reenen et al. [53] reviewed many previous studies and supported this issue. There were no significant associations between physical capacity tests, i.e., trunk muscle strength, muscle endurance, or mobility of the lumbar spine assessments, and the prevalence of LBP. Therefore, the relationship between PFT and the risk of MSDs remains inconclusive. PFT might be suitable for other types of workers rather than office workers and future studies on this topic should be conducted.

Our study encountered several limitations. Firstly, the cross-sectional survey conducted might have caused selection bias in this study. However, the 18 office workers who were unable to complete the PFT did not differ significantly on baseline characteristics from those who completed PFT (*n* = 545). Secondly, recall bias might have occurred in this study resulting in nonsignificant associations regarding many factors. However, MSDs were measured by asking about the past seven days to minimize recall bias and error. The online questionnaire provided descriptions and pictures to ensure participants’ understanding and promoted accurate responses. The OSCWE was available to answer via smartphone or computer and also reduced time to administer which could have increased the response rate among office workers.

## Conclusion

This study demonstrated the prevalence rate of MSDs and its associated risk factors among office workers. The risk factors associated with MSDs included BMI > 27.5 kg/m^2^, weight gain within two years, having comorbidity, MVPA ≥ 150 min/week and sitting at work ≥ 4 h/day, high to severe stress levels and sleep duration < 6 h/day. Our findings provide information to develop health promotion guidelines for Thai office workers. Specifically, office workers who have prolonged sitting at work should reduce sitting time and take frequent and short active breaks such as standing or walking every hour. Physically active office workers (MVPA ≥ 150 min/week) should take a break from prolonged sitting at work to prevent MSDs, NCDs and CMRFs. Daily balanced behavior between PA and SB should be considered. Secondly, although our findings did not identify a relationship between physical fitness tests and MSDs among office workers, the PFT might be appropriate for physical demanding workers rather than office workers. However, office workers should maintain physical capacity by being physically active and exercise, particularly those not meeting the PA recommendations and sitting for long periods. Third, office workers should avoid psychosocial and physical stress in the workplace. The company should set a policy of stress relief programs among office workers not only for stress management at work. They also have to manage time for good quality sleep for coping with stress and reducing risks of MSDs.

## Data Availability

The datasets generated and/or analyzed during the current study are not publicly available due to organizational confidential but are available from the corresponding author on reasonable request.

## Abbreviations

MSDs: Musculoskeletal disorders
PA: Physical Activity
SB: Sedentary Behavior
BMI: Body Mass Index
SPST-20: Suanprung Stress Test-20
MVPA: Moderate to Vigorous intensity Physical Activity
OSCWE: Online Self-reporting questionnaire on Computer Work-related Exposure
CCFT: Cranio-Cervical Flexion Test
DNF: Deep Neck Flexor
PAR-Q: Physical Activity Readiness Questionnaire
WC: Waist Circumference
BIA: Bioelectrical Impedance Analysis
HGS: Hand Grip Strength
DCF: Deep Cervical Flexor
PSIS: Posterior Superior Iliac Spine
THB: Thai Baht
